# Human-computer interaction emotional design and innovative cultural and creative product design

**DOI:** 10.3389/fpsyg.2022.982303

**Published:** 2022-09-27

**Authors:** Zhimin Gao, Jiaxi Huang

**Affiliations:** College of Fine Arts and Design, Huaihua University, Huaihua, China

**Keywords:** cultural and creative products, data mining, cluster analysis, emotional design, human-computer interaction

## Abstract

To make the interface design of computer application system better, meet the psychological and emotional needs of users, and be more humanized, the emotional factor is increasingly valued by interface designers. In the design of human-computer interaction graphical interfaces, the designer attaches great importance to the emotional design of the interface, and enhances the humanized design of the interface, which cannot only improve the comfort of the interface, but also improve the fun of the interface, to ensure the psychological and emotional needs of users can be better satisfied. It may acquire information that is favorable to innovative design by utilizing cluster analysis algorithm to tackle the problem of complicated cultural information, and then utilize cellular genetic algorithm to carry out creative design of cultural items. It increases the availability of cultural and creative goods. The classic cluster analysis technique offers the maximum data clustering effect of 53.3%, according to the findings of this paper’s experiments. While the improved cluster analysis algorithm has the highest data clustering effect of 90%. It can be seen that the improved cluster analysis algorithm can effectively perform cluster analysis on a large amount of data in cultural and creative products. It thus finds out the most suitable designer’s creative information, which helps designers create better products.

## Introduction

In the wave of the country’s vigorous development of the cultural industry, the competition of cultural product design is becoming more and fiercer, and the problem of homogeneity of product types and styles is becoming increasingly obvious. Innovation has become the key to winning the market. The consumption upgrade in today’s society has made consumers increasingly cautious in purchasing behavior. People are no longer limited to the needs of product functions, but focus on the meaning of products. Designers should meet the spiritual needs of consumers. This is also an academic circle. Major problems are faced. In the process of the development of Chinese traditional culture, regional culture has played a symbolic role, and the regional culture formed and accumulated from generation to generation in various places shows its unique spiritual charm. It is the brilliance of regional culture that forms the splendor of traditional Chinese culture. Specifically, in the design and development of cultural and creative products, over the years, people have often paid attention to and embodied the three main design elements of functionality, aesthetics, and culture, while ignoring the deeper emotional elements. The “user experience” often mentioned in product design actually refers to the “user emotional experience.” A good user experience comes from perfect function, suitable aesthetics, and profound culture. From an emotional point of view, function, aesthetics, these three points of culture serve emotion and form a causal relationship with the emotion elements, which is an important condition to complete emotion, which is a high-level information transmission process.

With the prosperity and development of social networks, communities, forums, etc., while conducting network activities, people are more inclined to share product designs and show their innovations on the Internet. This also produces a large amount of Web data, and the network value has reached an unprecedented level. There is a lot of valuable knowledge in these data, and only relying on experienced designers to complete the processing of these data, enterprises will be submerged by the wave of informatization. And Web data mining for product innovation service can make enterprises grasp design information more quickly and accurately. This makes the research on Web data mining methods for product innovation services urgent and more realistic. The innovation of this paper is that it proposes a data mining method to effectively process these information data. It integrates regional culture into the innovative design of cultural and creative products, to help designers better creative design.

## Related work

As people’s economic level is getting higher and higher in recent years, people’s demand for spirituality has also become increasingly. Cultural and creative products, namely, “cultural creative products,” refer to relying on the wisdom, skills, and talents of creative people. It creates cultural resources and stationery by means of modern technology. Wang found that cultural creativity has received much attention in recent years. He determined how to apply the best of it to commercial products, requiring the investigation and compilation of large amounts of cultural information to develop new cultural products (Wang, 2018). Naquin S explored the cultural material products that most people experience the world. People will not only understand and pay attention to these regional cultures, will open up new research fields, but also historians will be more capable of evaluating cultural and creative products (Naquin, 2018). A Vives-Riera provided a microhistorical approach to the formation of regional cultures in the 19th and 20th centuries. In the process of regionalization of music, local musicians strategically use cultural authority about the island to gain a certain power, negotiating their own regional identity from below to nationalized agencies (Vives-Riera, 2018). Ode L used interviews, observations, and recordings to collect data on regional cultures. The results show that modern society has rich cultural treasures, to protect this cultural heritage, efforts need to be protected, developed, and utilized (Ode et al., 2021). To achieve regional industrial revival, Bi W relied on the development of cultural tourist resources and the growth of the cultural tourism sector. He presented a strategy based on big data and the Internet of Things for local IP creation and cultural creative design, as well as data cleansing and processing *via* the Internet of Things (Bi and Wang, 2021). Li L aimed to enhance the enthusiasm of creative designers and promote the coordination of regional culture and cultural and creative products. He constructed a creative incentive model for the cultural and creative industry chain (Li and Gao, 2019). Scholars all believe that regional culture and cultural and creative products should be combined, but there are no specific measures.

People’s lives are surrounded by data mining technology. According to Xu L, the growing popularity and advancement of data mining technologies has resulted in major concerns on personal information security. Privacy-preserving data mining (PPDM) has received a lot of attention in recent years. He also investigated alternative strategies for safeguarding sensitive data (Xu et al., 2017). Even after considering data mining, Yan X S discovered that several basic signals are important predictors of market performance (Yan and Zheng, 2017). Data mining can be traced back to exploratory data analysis, according to Slater S. He went over some of the new tools that are being developed for educational data mining research and practice. The broader study fields of data mining and data science employ discussion-related methods as well (Slater et al., 2017). Although scholars have found that data mining can be applied in many fields, they have not combined data mining with cultural and creative products.

## Information processing and innovative design methods for cultural and creative products based on data mining

### Processing of product information based on data mining clustering algorithm

The essence of product innovation is knowledge innovation, and the design of innovative products needs to be based on solid knowledge accumulation. It perfectly combines excellent design concepts with products. This requires designers to continuously accumulate in learning, continuously improve their own aesthetics, improve self-cultivation, track and predict the market trend, and gain insight into the real needs of life. In the final analysis, it is the process of knowledge discovery and processing (Chen et al., 2021).

Behind a series of buzzwords such as big data accurate marketing and big data insight, data mining and analysis technology plays an important role in various industries. Although the amount of information on the Web is large, it can be described as massive. However, there is very little information that meets the needs of users, and people are not satisfied with just using the Web as a platform for information release and information sharing. How to obtain valuable knowledge and hidden information by mining Web documents and services, and provide information services that facilitate people’s lives and work on this basis has become an urgent need for people. Web data mining is a technology produced in this context (Feng and Zhang, 2017).

#### Clustering algorithm based on cosine similarity

Cluster analysis is one of the main tasks of data mining. Moreover, clustering can be used as an independent tool to obtain the distribution of data and observe the characteristics of each cluster of data. It focuses on a specific set of clusters for further analysis. To examine the degree of similarity between a web page and a cluster, this paper applies the cosine similarity algorithm in the cosine similarity algorithm. The degree of similarity between the vectors is determined according to the angle between the two vectors. When the angle between the two vectors is 0 degrees, the two are in a parallel relationship, and the two vectors are considered the most similar (Wu and Peng, 2017). Cosine similarity, also known as cosine similarity, is to evaluate the similarity of two vectors by calculating the cosine value of the angle between them. According to the cosine similarity algorithm, the similarity between vectors is defined as Eq. 1:


(1) $$Sim_{\text{C}}\left(A_{\text{j}},C_{\text{j}}\right)=\frac{\sum_{k=1}^{\text{n}}a_{i,k}*c_{j,k}}{\left|A_{\text{j}}\right|*\left|C_{\text{j}}\right|}$$

In Web page vectors, the weights of keywords are different. With the process of clustering, more web pages will be clustered into cluster C_j_, which will cause the position of vector *C*_j_ to change, thus affecting the clustering effect. Equation 1 is transformed to obtain Eq. 2:


(2) $$Sim_{\text{C}}\left(A_{\text{j}},C_{\text{j}}\right)=\frac{\sum_{k=1}^{\text{n}}\frac{Na_{i,k}}{N_{\text{j}}}a_{i,k}*c_{j,k}}{\left|A_{\text{j}}\right|*\left|C_{\text{j}}\right|}$$

Na_i,k_ represents the number of *a*_i,k_ s in the k-th keyword of the Web page vector in cluster C_j_, and N_j_ represents the number of Web pages in cluster C_j_ (Myunggyo et al., 2017).

To derive different clusters, the calculation method is defined as Eq. 3:


(3) $$Sim_{\text{C}}\left(C_{\text{i}},C_{\text{j}}\right)=\frac{\sum_{k=1}^{\text{n}}\left(\frac{Nc_{i,k}}{N_{\text{i}}}c_{i,k}*\frac{Nc_{j,k}}{N_{\text{j}}}c_{j,k}\right)}{\left|C_{\text{i}}\right|*\left|C_{\text{j}}\right|}$$

N_i_ and N_j_ represent the *i*-th and *j*-th clusters, respectively, and Nc_i,k_, Nc_j,k_ represents the number of web pages with the *k*-th keyword/item value other than 0 in the *i*-th and *j*-th clusters (Lin et al., 2018).

#### Improvement based on cosine similarity algorithm

By observing Eq. 1, it can be found that when calculating the similarity between a web page and a cluster, each keyword/item appearing in the web page is a key parameter that affects the similarity between the two. Therefore, in the clustering process, each keyword should play a role under a separate parameter, so the Jaccard similarity criterion is introduced (Moustakas, 2017). It is used to compare similarities and differences between limited sample sets, such as the similarity between sets, the similarity of strings, and the similarity of target detection.

To calculate the effect of each keyword under separate parameters, the Jaccard similarity criterion for two vectors *a* and *b* with coefficients of 0 or 1 is defined as Eq. 4:


(4) $$Sim\left(a,b\right)=\frac{\#\left(a_{\text{i}}=b_{\text{i}}=1\right)}{\#\left(a_{\text{i}}=1\right)+\#\left(b_{\text{i}}=1\right)-\#\left(a_{\text{i}}=b_{\text{i}}=1\right)}$$

Based on the above, applying the Jaccard similarity criterion under each keyword, the similarity calculation method between a web page vector and the considered cluster vector is summarized as Eq. 5:


(5) $$Sim_{\text{j}}\left(A_{\text{j}},C_{\text{j}}\right)=\frac{\sum_{k=1}^{\text{n}}a_{i,k}*c_{j,k}}{\sum_{k=1}^{\text{n}}a_{i,k}^{2}+\sum_{k=1}^{\text{n}}c_{j,k}^{2}-\sum_{k=1}^{\text{n}}a_{i,k}*c_{j,k}}$$

Combining Eqs 4, 5, the similarity algorithm matrix between web pages and clusters is corrected as shown in Eq. 6:


(6) $$Sim_{\text{JC}}\left(A_{\text{j}},C_{\text{j}}\right)=Sim_{\text{j}}\left(A_{\text{j}},C_{\text{j}}\right)*Sim_{\text{C}}\left(A_{\text{j}},C_{\text{j}}\right)$$

The similarity calculation method between clusters under the Jaccard similarity criterion is as shown in Eq. 7:


(7) $$Sim_{\text{j}}\left(A_{\text{j}},C_{\text{j}}\right)=\frac{\alpha }{\sum_{k=1}^{\text{n}}c_{i,k}^{2}+\sum_{k=1}^{\text{n}}c_{j,k}^{2}-\alpha }$$

Combining Eqs 6, 7, the similarity between the two clusters based on the Jaccard cosine similarity algorithm is defined as shown in Eq. 8:


(8) $$Sim_{JC}\left(C_{\text{i}},C_{\text{j}}\right)=Sim_{\text{j}}\left(C_{\text{i}},C_{\text{j}}\right)*Sim_{\text{C}}\left(C_{\text{i}},C_{\text{j}}\right)$$

In the Web data mining clustering algorithm proposed in this paper, to obtain the optimal cluster, only the cluster with the largest similarity is merged. And under certain conditions, the optimization operation of clustering is performed. The advantage of the algorithm proposed in this paper is that when creating clusters, the Jaccard similarity criterion and the cosine similarity algorithm are used comprehensively.

In practical applications, the results produced by clustering algorithms may vary greatly in datasets from different fields. The output of the algorithm is closely related to the settings of some important parameters such as the number of clusters, initial cluster centers, etc. Inappropriate algorithm selection and parameter settings may lead to wrong conclusion that deviate from reality (Chaurasia and Pal, 2017).

**FIGURE 1:**
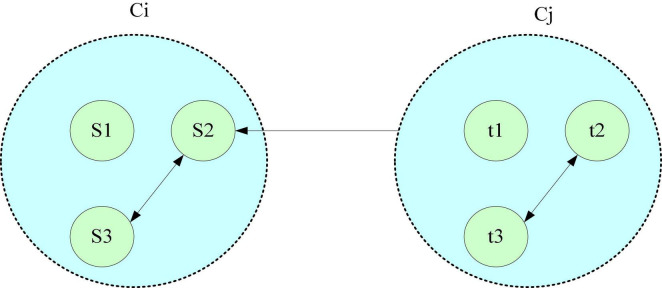
Diagram of relationships between clusters.

The purpose of evaluating a clustering algorithm is to find the clusters that have the best agreement with the data under consideration. The two basic criteria for evaluating and selecting a clustering algorithm are: the compactness within the same cluster and the separation between different clusters. The evaluation of clustering results is generally divided into two types: external evaluation criteria and internal evaluation criteria (Abedallah and Anqi, 2022). The relationship between clusters is shown in Figure 1.

As shown in Figure 1, the Dunn Index is the quotient of the shortest distance between the sample points of any two clusters and the maximum distance of the sample points in any cluster. The larger the value, the better the clustering effect. The clustering evaluation method used in the paper is based on the internal evaluation standard Dunn Index, which is used to obtain the optimal number of classifications. Let P be the initial web page cluster, and the distance between two web page vectors or two cluster vectors is as Eq. 9:


(9) Dis tan⁡ce=1-Similarity

Among them, similarity is the similarity between two web page vectors or two cluster vectors, including two types of similarity based on cosines and similarity based on Jaccard cosine.

The cluster diameter is the maximum distance between the web page vectors included in the cluster, and its calculation method is as shown in Eq. 10:


(10) $$Diam_{\text{i}}=\max\left\{Dis\;\tan ce\left(a,b\right)|a,b\in C_{\text{i}}\right\}$$

The intercluster distance is the minimum distance between all web page vectors in one cluster and all web page vectors in another cluster.

**FIGURE 2:**
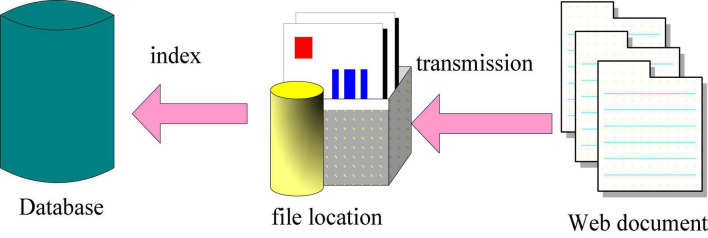
Structure diagram of product innovation information cache.

Without the assistance of the product innovation information cache library, each time the system runs, the database-related operations are completed at the same time, and then the mining can be carried out. This seriously affects the running speed of the data mining system. By building a product innovation information cache library, this problem can be well solved. According to the needs of product innovation, relevant pages, documents, and other information are obtained from specific keywords, and these information are downloaded and saved to the product innovation information cache database. It further greatly improves the workload of the mining system per unit time, and its structure is shown in Figure 2.

As shown in Figure 2, the realization method is to open up a disk space on the computer to store the information related to product innovation obtained from the network. It sets the storage path of the network information acquisition results, and saves the product innovation Web pages and documents obtained from the network to the cache information database. By indexing the obtained documents, it can be easily processed by the mining system (Mueller et al., 2018).

### Creative design based on cellular genetic algorithm

The production of the scheme in traditional product modeling design is mostly dependent on the designer’s creative ideas. Excellent designers must possess a specific range and depth of knowledge structure, as well as active perceptual and rational thinking. A unique design method for product modeling based on cellular genetic algorithm and standard genetic algorithm is proposed to fulfill the different emotional needs of users and to simulate the initial design and detailed design in design thinking.

Cellular Genetic Algorithm is a heuristic optimization algorithm that can simulate the biological world and have human-computer interaction experience. To a certain extent, it can simulate the design thinking of designers.

**FIGURE 3:**
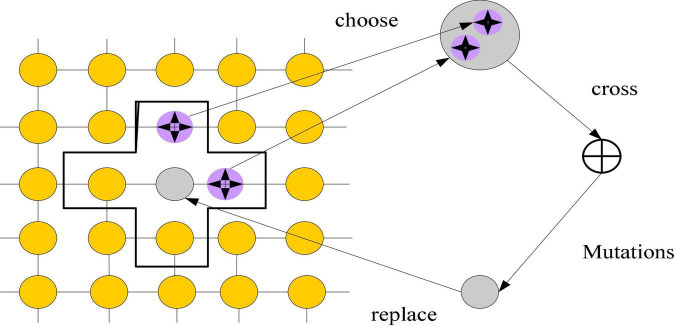
Cell operation flow.

The cellular genetic algorithm can do relatively fast global optimization based on the central cell, which can better imitate the initial design thinking in product innovation design. The algorithm’s execution procedure involves mapping evolutionary individuals into a topological framework. Individual genetic procedures are restricted to their appropriate communities. The global optimal solution is found through the interaction of local individuals, according to a certain updating approach. It has been used in many fields (Bian, 2020; Dai, 2022) and cannot only sustain population variety but also has an excellent spatial search capacity. Figure 3 depicts the cell operation process.

**FIGURE 4:**
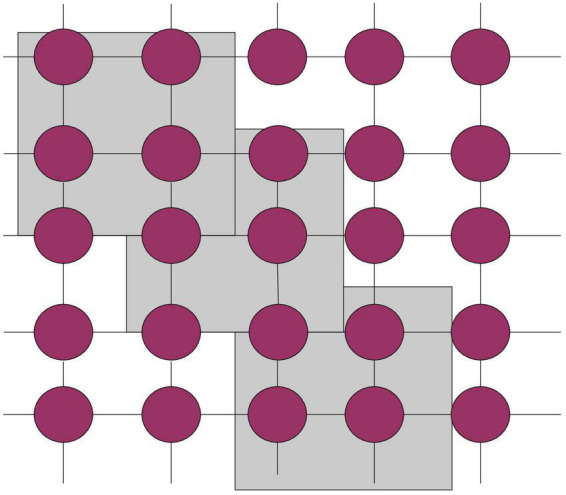
Linear sweep.

As shown in Figure, the selection of the update strategy in the cellular genetic algorithm can influence the genetic operations in several sectors. The linear scan updating approach is used in this work, and cell operations are carried out in the unit comprising the core cell and its neighbors. Cellular automata are not determined by strictly defined physical equations or functions, but are composed of rules constructed by a series of models. Any model that satisfies these rules can be regarded as a cellular automata model. Figure 4 shows the linear scan.

As shown in Figure 4, the most commonly used method in the update strategy of the cellular genetic algorithm is linear scanning, that is, the cell operations are performed on the cells in the grid in units of units. It uses linear scanning technology, and when the inspection object passes the camera, the camera will build an image for each pixel line. When simulating cells in 1950, cellular automata was initially proposed, and it lies at the heart of the cellular genetic algorithm. It is made up of four basic components: a cell, cell space, neighbors, and evolution principles like Eq. 11:


(11) $$A=\left(L_{\text{d}},S,N,f\right)$$

Among them: A represents cellular automata; L_d_ represents the cellular space, d represents the dimension of the cellular space.

The triangular grid has certain limitations due to its small number of neighbors, the hexagonal grid is difficult to express in computer language due to its complexity, and the square grid has a moderate number of neighbors and is easy to implement with the aid of a computer.

Von Neumann type, Moore type, extended Moore type, and other nearby bodies are examples. Both the cell and its current neighbor structure can determine the state of the core cell at the next moment for different neighbor structures. In computational theory, Moore machines are finite state machines whose output values are determined only by their current state. The neighbors of the central cell are the four cells to the east, south, west, and north. The radius of a neighbor is 1, as specified by Eq. 12:


(12) $$N_{\text{Neumann}}=\left\{v_{\text{i}}=\left(v_{\text{i}}a,v_{\text{i}}b\right)+|v_{\text{oa}},v_{\text{ob}}|\right\}$$

(*v*_i_*a*, *v*_i_*b*) represents the row and column coordinate value of the neighbor cell.

The neighbor radius is 1, which is defined as Eq. 13:


(13) $$N_{M\text{oo}\mathrm{ne}}=\left\{v_{\text{i}}=\left(v_{\text{i}}a,v_{\text{i}}b\right)|v_{\text{i}}a-v_{ob}|\leq 1\right\}$$

The extended Moore type means that the neighbor radius extends to 2 and above, and its neighbor is defined as Eq. 14:


(14) NMoone={vi=(via,vib)|via-vob|+|bi-vob|≤r}

Evolutionary algorithm, also known as evolutionary algorithm, evolutionary computation, or genetic algorithm. The cellular neighbor model replaces the single cell in the first three neighbor models with a 2 × 2 cell block. Therefore, it is necessary to clarify the neighbor structure of the cell to pave the way for the cell operation and update strategy. The dynamic state of an individual is determined by evolutionary rules, and the interaction and mutual influence of genetic operations among neighborhoods are completed by an update strategy. The update strategy refers to the way that each neighborhood cell operation is performed in the cellular genetic algorithm, that is, the way that each cell neighborhood interacts and influences each other.

The evolution rule is the main component of the cellular genetic algorithm, which can simulate natural phenomena such as birth, aging, sickness, and death, and determine the dynamic function of the cell state at the next moment according to the current state of the cell and its neighbors. At the same time, the states of the central cells will influence each other and promote the interaction between local populations. Its basic model is Eq. 15:


(15) $$S^{t+1}=\left\{ \begin{array}{c} 1,A_{\text{i}}\leq n\leq B_{\text{i}}\\ 0,n\geq A_{\text{j}},n≻B_{\text{j}} \end{array}\right.$$

Among them, *S*^t + 1^ represents the state of the cell at time *t* + 1, and for the “live” central cell (corresponding to 1), A_i_,B_i_ represents the number of “live” neighbors needed to maintain its state at the next moment. For the “dead” central cell (corresponding to 0), A_i_,B_i_ represents the number of “dead” neighbors that need to keep its state at the next moment.

**FIGURE 5:**
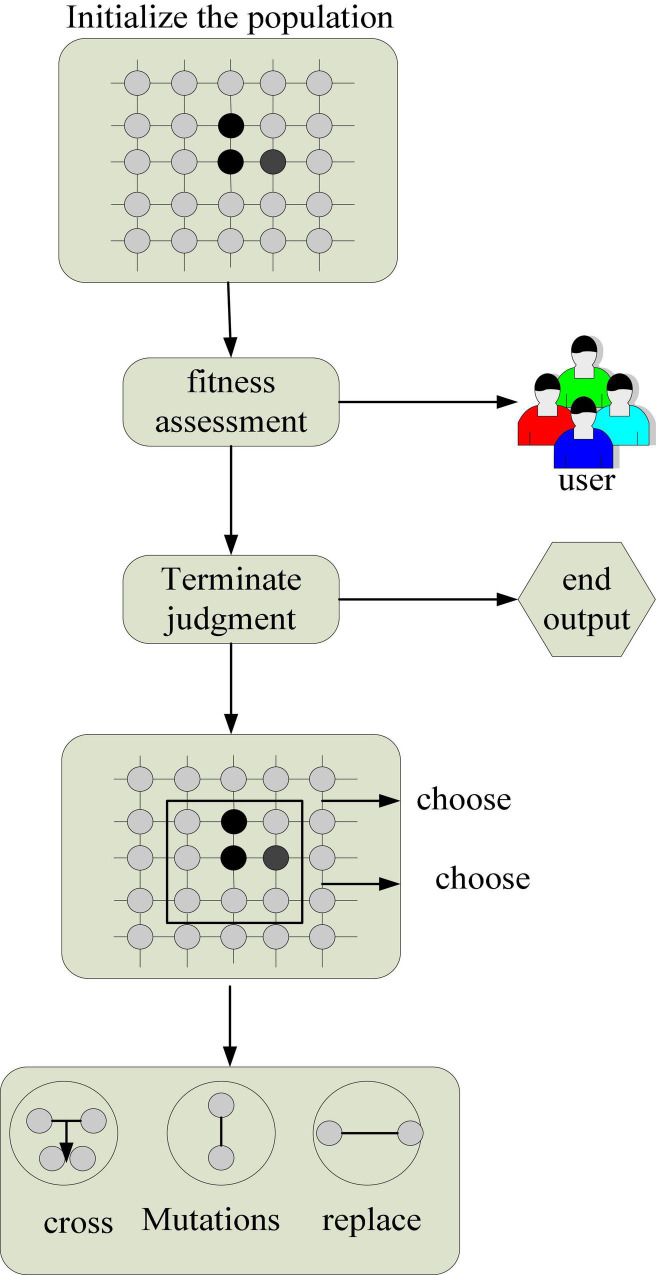
Product innovation design process.

This study examines the early design process of computer-aided product modeling innovation that follows the law of design thinking activities, starting with the inspiration of designers’ natural inventive thinking. It provides a theoretical foundation for developing a system for product modeling, innovation, and refinement. Figure 5 depicts the early design process for product modeling innovation using a cellular genetic algorithm.

As indicated in Figure 5, this research examines the early design steps of product modeling innovation. It synthesis and evaluation as the basic unit of the design process, and takes divergent and convergent thinking as the core of the design thinking mode. Its design strategy structured by breadth-first, depth-first, and opportunism is a common principle for designers to focus on design solutions. It mainly searches for product opportunity gaps by dominating divergent thinking and guided by a breadth-first strategy.

In terms of thinking and process, divergent and convergent thinking are complementary, and genetic algorithms have better convergence. As a result, the novel design of product modeling is investigated using the convergence features and convergence thinking of genetic algorithms. Global convergence refers to whether the algorithm converges when the initial value is arbitrarily selected within the definition domain. Equation 16 defines the evolutionary algorithm’s global convergence:


(16) $$Z_{\text{t}}=\max\left\{f\left(\prod_{K}^{\left(t\right)}\left(i\right):K=1,2,\ldots ,n\right)\right\}$$

If and only if


(17) $$\lim P\left\{Z_{\text{t}}=f^{*}\right\}=1$$

Among them:


(18) $$f^{*}=\max\left\{f\left(b\right)|b\in IB^{l}\right\}$$

Equation 18 is used to ensure that the evolutionary algorithm eventually finds the best solution set. When the product shape fulfills the user’s needs and the quantity achieves the preset value in evolutionary algebra, the individual with the highest fitness achieved during the evolution process is used as the optimal solution output, and the operation is completed. The maximum evolutionary algebra is the set number of loops.

If the new product shape is better than the original shape, the gene of the central cell is replaced with the new product shape gene. Otherwise, it keeps the original central cell gene unchanged. The replacement operation *l*_*vox*_ is defined as Eq. 19:


(19) $$l_{vox}=l_{vox}^{t+1}$$

It decodes and displays the new product shape. If there is a satisfactory solution, it is selected as the initialized individual for the detailed design; otherwise, the above operations are repeated until a satisfactory conceptual design solution is obtained.

### Development of cultural and creative products based on regions

The homogeneity of the territory itself is the definition of boundaries according to specific characteristics. In the research field of this topic, the regional characteristics are mainly described by single or several characteristics. From a professional point of view, regionality should include unique regional visual elements composed of regional natural characteristics, architectural decoration, human habits, and specific regional culture. The regional culture of a country refers to the totality of regional cultural values and appearances presented in material and spiritual aspects of different regions. Regional culture includes collections of history, literature, art (modern art, traditional crafts), folklore, science, and technology, etc., which show the wide and far-reaching origin of culture.

The cultural creation industry was once considered one of the most promising industries in the world in the 21st century. From the late 1990s to the present, cultural and creative industries have developed rapidly in the world. Developed countries have shown relatively mature model systems. Since the beginning of the 21st century, the Chinese government has successively issued a series of policies on cultural and creative industries, actively supporting the development of cultural and creative industries. It regards the development of culture and creative industries as the new economic growth point of people’s national economy. With the continuous improvement of the government’s policies on cultural and innovative industries, the cultural and innovative industries in China’s important economic and cultural center cities are also flourishing.

As shown in cultural and creative products can be said to be the last “exhibition area” to visit the Mausoleum of Qin Shihuang, and cultural and creative products with the function of disseminating culture must inevitably incorporate cultural elements. In Xi’an, the most famous tourist product is the imitation model of the “Terracotta Warriors and Horses” of the Mausoleum of Qin Shihuang. Its shape has profound Qin Dynasty cultural accumulation and rich research value. The regional culture it shows has a history of thousands of years. The tourism series products on the theme of “Terracotta Warriors and Horses” are quite rich in form and content, such as stamps, painted sculptures, clay, pottery, T-shirts, key chains, and other peripheral products, all of which reflect the diversity of regional culture in the brand image design of tourism products.

## Experiment and innovative design of cultural and creative products based on data mining

### Experiment of cluster effect based on data mining

This experiment conducts experiments and comparisons on five datasets, which are Iris, Wine, glass, Zoo, and Balance-Scale. The first to fifth sets of data use the datasets in the university of california lrvine (UCI) Machine Learning Repository. In this experiment, the traditional clustering analysis algorithm is given and the clustering effect of the improved clustering algorithm will be compared. The three algorithms are run 30 times in each data set. The comparison is shown in Tables 1, 2.

**TABLE 1:** Clustering effects of traditional clustering analysis algorithms.

Data set	Number of runs	Number of clusters	Clustering efficiency
Iris	30	15	50.0%
Wine	30	16	53.3%
Glass	30	10	33.3%
Zoo	30	12	40.0%
Balance-Scale	30	13	43.3%

**TABLE 2:** Clustering effect of the improved clustering analysis algorithm.

Data set	Number of runs	Number of clusters	Clustering efficiency
Iris	30	27	90.0%
Wine	30	23	76.7%
Glass	30	24	80.0%
Zoo	30	25	83.3%
Balance-Scale	30	26	86.6%

As shown in Tables 1, 2, the highest number of clusters is 16 in the 30 runs of the traditional cluster analysis algorithm. The clustering efficiency is 53.3%, the minimum number of clusters is 10, the clustering efficiency is 33.3%, and the general clustering efficiency is low. In the improved cluster analysis algorithm, the highest number of clusters is 27, the clustering efficiency is 90.0%, the lowest number of clusters is 23, the clustering efficiency is 76.7%. Overall, the clustering effect is very high, it can be seen that the effect of the improved clustering analysis is better.

The paper uses the same dataset to simulate under different similarity algorithms. The two similarity algorithms are the algorithm based on cosine similarity and the algorithm based on Jaccard cosine similarity, and the results obtained from the simulation are evaluated by Dunn Index. The results after evaluation are shown in Tables 3, 4.

**TABLE 3:** Evaluation results of algorithms based on cosine similarity.

Data scale	Minimum distance between clusters	Maximum diameter within a cluster	Dunn Index
200	0.565	0.984	0.574
400	0.406	0.989	0.410
600	0.406	0.993	0.408
800	0.304	0.993	0.306
1000	0.304	1.000	0.304

**TABLE 4:** Evaluation results of algorithms based on Jaccard cosine similarity.

Data scale	Minimum distance between clusters	Maximum diameter within a cluster	Dunn Index
200	0.661	0.841	0.786
400	0.523	0.900	0.581
600	0.502	0.938	0.536
800	0.416	0.943	0.441
1000	0.400	0.964	0.415

**FIGURE 6:**
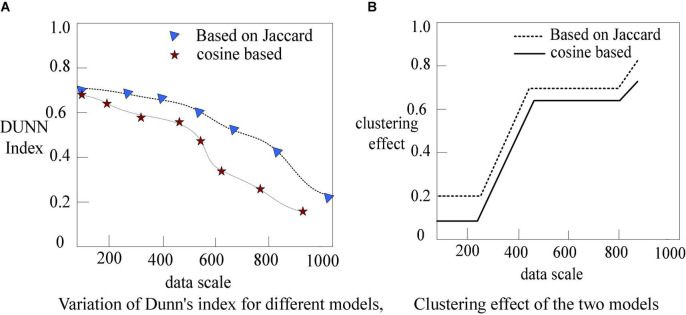
Simulation results of Dunn’s index as an evaluation criterion. **(A)** Variation of Dunn’s index for different models. **(B)** Clustering effect of the two models.

As shown in Tables 3, 4, this paper compares the results obtained on the same data scale. The minimum distance between clusters obtained by the algorithm based on cosine similarity in Web data mining clustering is always smaller than the algorithm based on Jaccard cosine similarity. The maximum diameter within clusters obtained by the algorithm based on cosine similarity is always larger than that obtained by the algorithm based on Jaccard cosine similarity. The simulation results of this paper using Dunn Index as the evaluation standard are shown in Figure 6.

As shown in Figure 6, in the comparison of the Dunn Index calculated by the formula, with the increase of data size, the Dunn Index of the algorithm based on Jaccard cosine similarity is at a higher level than that of the algorithm based on cosine similarity. Through the above analysis, we can see that the Web data mining clustering algorithm based on Jaccard cosine similarity algorithm proposed in this paper can perform better clustering.

### Experiment based on genetic algorithm

**FIGURE 7:**
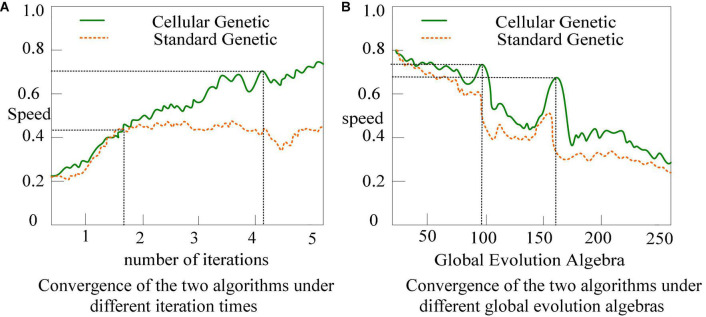
Changes in the convergence of standard genetic algorithm and cellular genetic algorithm. **(A)** Convergence of the two algorithms under different iteration times. **(B)** Convergence of the two algorithms under different global evolution algebras.

Each dataset has different dimensions. Therefore, to unify and prevent it from unnecessarily affecting the experimental results, when using any data set for experimental testing and verification, people first perform standard normalization on all data sets. In this paper, *Z*-Score data standardization technology is used. *Z*-Score normalization is a common method of data processing. Through it, data of different magnitudes can be converted into a unified measure of *Z*-Score for comparison. It improves data comparability. And the convergence of standard genetic algorithm and cellular genetic algorithm is shown in Figure 7.

The normal genetic algorithm’s convergence pace is substantially slower than the cellular genetic algorithm’s, as shown in Figure 7, and the algorithm geometry begins to converge slowly after 150 generations.

It also illustrates the high-efficiency characteristics of the cellular genetic algorithm from another aspect. Of course, it also illustrates that it is easy to fall into the local optimum when there are multiple local optimal solutions in the data set. The standard genetic algorithm can search from the problem solution space in a wide range, but it lacks the ability of local development, and the convergence of large data sets is very slow, which is a waste of time. This research employs the conventional genetic algorithm as the global search algorithm and the cellular genetic algorithm as the local search algorithm to verify that the cellular genetic algorithm may increase the algorithm’s performance.

**FIGURE 8:**
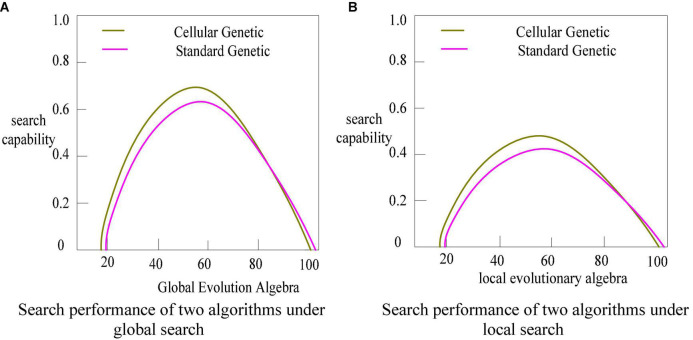
Comparison of search performance between standard genetic algorithm and cellular genetic algorithm. **(A)** Search performance of two algorithms under global search. **(B)** Search performance of two algorithms under local search.

To compare the search performance of the two on the dataset, this article records the best search result achieved by each algorithm iteration as well as the current number of iterations. Standard Genetic Algorithms mostly use the binary coding method, and the decision variables are represented by binary strings, and the length of binary coding is determined by the required precision. Figure 8 depicts the outcomes: the normal genetic algorithm’s convergence pace is substantially slower than the cellular genetic algorithm’s.

As shown in Figure 8, the cellular genetic algorithm’s search speed is pretty quick, and it tends to converge within around 60 generations, whereas the normal genetic algorithm’s search pace is relatively slow. When comparing the two, not only in terms of performance, the cellular genetic algorithm outperforms the classic standard genetic algorithm.

## Conclusion

By increasing the investment in emotional design in cultural and creative products, it cannot only realize the important function of “cultural people,” but also make cultural and creative products form a unique user experience, avoid the phenomenon of homogeneity, and highlight the uniqueness, through the emotional capture of consumer groups, it can effectively segment the market, provide more accurate services, and lay a good foundation for the development of the cultural and creative industry to a higher level. With the development of science and technology, the needs of users have extended from functional needs to psychological needs. It needs to integrate humanistic feelings such as individual style, self-demand, and aesthetic expression into the creative design of cultural products. And the products with personalized services are now becoming the object of increasing attention of designers. The designed products not only need to realize the user function, but also realize it in terms of emotional and customized demands. As a result, this article put forward developing novel cultural and creative product based on regional culture. A clustering analysis algorithm based on data mining is proposed in light of the vast amount of data in the inventive design of cultural and creative items in today’s information network. The system successfully enhances data processing speed and cultural information grouping, allowing designers to expand their creative space. The cellular genetic algorithm generates cultural creative design ideas. This study compares the performance of the two algorithms in the experimental section. The results reveal that the improved clustering analysis algorithm clusters better than the traditional clustering algorithm, and that the cellular genetic algorithm has superior search ability than the regular genetic algorithm. However, because the experimental environment is affected by external factors, there are still differences in the data of the experimental part.

## Data availability statement

The original contributions presented in this study are included in the article/supplementary material, further inquiries can be directed to the corresponding author.

## Author contributions

Both authors have participated in conception and design, or analysis and interpretation of the data, drafting the manuscript, or revising it critically for important intellectual content and read and approved the final manuscript.
